# ΔFosB Decreases Excitability of Dorsal Hippocampal CA1 Neurons

**DOI:** 10.1523/ENEURO.0104-18.2018

**Published:** 2018-08-03

**Authors:** Andrew L. Eagle, Elizabeth S. Williams, Joseph A. Beatty, Charles L. Cox, Alfred J. Robison

**Affiliations:** Department of Physiology and Neuroscience Program, Michigan State University, East Lansing, Michigan 48824

**Keywords:** ΔFosB, CA1, excitability, hippocampus, transcription factor

## Abstract

Both the function of hippocampal neurons and hippocampus-dependent behaviors are dependent on changes in gene expression, but the specific mechanisms that regulate gene expression in hippocampus are not yet fully understood. The stable, activity-dependent transcription factor ΔFosB plays a role in various forms of hippocampal-dependent learning and in the structural plasticity of synapses onto CA1 neurons. The authors examined the consequences of viral-mediated overexpression or inhibition of ΔFosB on the function of adult mouse hippocampal CA1 neurons using *ex vivo* slice whole-cell physiology. We found that the overexpression of ΔFosB decreased the excitability of CA1 pyramidal neurons, while inhibition increased excitability. Interestingly, these manipulations did not affect resting membrane potential or spike frequency adaptation, but ΔFosB overexpression reduced hyperpolarization-activated current. Both ΔFosB overexpression and inhibition decreased spontaneous excitatory postsynaptic currents, while only ΔFosB inhibition affected the AMPA/NMDA ratio, which was mediated by decreased NMDA receptor current, suggesting complex effects on synaptic inputs to CA1 that may be driven by homeostatic cell-autonomous or network-driven adaptations to the changes in CA1 cell excitability. Because ΔFosB is induced in hippocampus by drugs of abuse, stress, or antidepressant treatment, these results suggest that ΔFosB-driven changes in hippocampal cell excitability may be critical for learning and, in maladaptive states, are key drivers of aberrant hippocampal function in diseases such as addiction and depression.

## Significance Statement

Memory consolidation is a key component of adaptive learned behavior, which can be disrupted in psychiatric conditions, and regulated gene expression in hippocampus is critical for learning and memory. ΔFosB is a highly stable, activity-dependent transcription factor that regulates hippocampus-dependent memory, yet its specific role in hippocampal neuronal function is unclear. The authors use whole-cell slice electrophysiology to uncover a novel role for ΔFosB in regulating neuronal excitability in the CA1 region of hippocampus. These findings suggest that ΔFosB-driven changes in gene expression drive the intrinsic excitability of hippocampal CA1 pyramidal neurons, potentially regulating learning, antidepressant responses, and psychiatric diseases such as drug addiction and depression.

## Introduction

Memory consolidation is indispensable to mammalian survival, and disruption of memory underlies numerous aberrant clinical conditions including neurodegenerative disorders, psychiatric disease, and neurodevelopmental impairments. The formation and retrieval of memories rely on changes in gene expression, and activity-dependent gene transcription in the hippocampus (HPC), in particular, is necessary for long-term memory consolidation and normal HPC function (Alberini and Kandel, 2014; Eagle et al., 2016). Transcription underlies durable modifications in HPC neuronal physiology, including synaptic and nonsynaptic plasticity (Guzman-Karlsson et al., 2014), and it is well established that pharmacological blockade of transcription impairs long-term plasticity and memory consolidation (Squire and Barondes, 1970; Frey et al., 1996; Lee et al., 2004). Given that this process is important for HPC function and learning, it is critical that we fully delineate the mechanism by which activity-dependent changes in gene transcription drive altered neuronal physiology.

ΔFosB is a unique activity-dependent transcription factor that is remarkably stable, with a half-life of up to 8 d in the brain (Carle et al., 2007; Ulery-Reynolds et al., 2009; Cates et al., 2014), thereby making it an interesting candidate for regulation of gene transcription in long-lasting processes such as memory consolidation. Hippocampal ΔFosB is important for learning and CA1 neuron structural plasticity (Eagle et al., 2015; Corbett et al., 2017). ΔFosB is induced specifically in dorsal HPC (dHPC) CA1 pyramidal neurons during spatial learning, and its transcriptional activity in those cells is crucial for multiple HPC-dependent forms of learning and memory. However, the physiologic mechanism by which ΔFosB regulates hippocampal neuronal function remains unknown.

ΔFosB differentially regulates synaptic strength in dopamine D_1_ and D_2_ receptors expressing medium spiny neurons (MSNs) of the nucleus accumbens (NAc) in a subregion-specific manner (Grueter et al., 2013). These findings suggest that ΔFosB induces cell type-specific plasticity in NAc and that this may provide a novel target for the treatment of addiction and depression. ΔFosB in prefrontal cortex may also regulate projection-specific cell function, and this may underlie depressive and/or post-traumatic stress disorder phenotypes (Vialou et al., 2014). Because its role in HPC cell function remains unknown, we investigated the role of ΔFosB in membrane properties, excitability, and synaptic function in dHPC neurons. We used viral-mediated gene transfer to overexpress ΔFosB or silence its transcriptional activity specifically in dorsal CA1 neurons *in vivo* and examined cell properties *ex vivo* using slice whole-cell electrophysiology. We report here that ΔFosB reduces neuronal excitability in CA1 neurons of the dHPC.

## Materials and Methods

### Animals

This study followed guidelines described in the *Guide for the Care and Use of Laboratory Animals* (Institute of Laboratory Animal Resources (U.S.), 2011). All animal procedures were performed in accordance with the regulations of the Michigan State University animal care committee. Adult male C57BL/6J mice (https://www.jax.org/strain/000664), 7–9 weeks of age, were group housed (*n* = 4–5/cage) in a vivarium and kept at 20–23°C under a 12 h light/dark cycle with *ad libitum* access to food and water.

### Viruses and surgery

Mice received viral infusions into the dHPC. Stereotaxic surgery was performed to inject herpes simplex virus (HSV) vectors into bilateral dHPC, as follows: 7° angle; −2.2 mm anteroposterior, ±2.0 mm ML, −2.0 and −1.8 mm DV (0.3 μl of purified virus was infused at each DV site at a rate of 0.1 μl/min). HSV expressing GFP, ΔFosB + GFP, or ΔJunD + GFP driven by the cytomegalovirus promoter were obtained from the Massachusetts Institute of Technology Viral Core Facility [now moved to Massachusetts General Hospital (https://researchcores.partners.org/mvvc/about)]. The approximate titer for all viruses used was ∼1.0 × 10^9^ virions/ml.

### Slice preparation

All solutions were bubbled with 95% O_2_-5% CO_2_ throughout the procedure. Mice were anesthetized with isoflurane and transcardially perfused with sucrose artificial CSF (aCSF; in mm: 234 sucrose, 2.5 KCl, 1.25 NaH_2_PO_4_, 10 MgSO_4_, 0.5 CaCl, 26 NaHCO_3_, and 11 glucose). Brains were rapidly removed, blocked, and placed in cold sucrose aCSF. Coronal sections (250 μm) containing dHPC were cut on a vibratome (Leica) and transferred to an incubation chamber containing aCSF (in mm: 126 NaCl, 2.5 KCl, 1.25 NaH_2_PO_4_, 2 MgCl, 2 CaCl, 26 NaHCO_3_, and 10 glucose) held at 34°C for 30 min before moving to aCSF at room temperature until used for recordings. Recordings were made from a submersion chamber perfused with aCSF (2 ml/min) held at 32°C.

### Electrophysiology

Borosilicate glass electrodes (3–6 MΩ) were filled with K-gluconate internal solution (in mm: 115 potassium gluconate, 20 KCl, 1.5 MgCl, 10 phosphocreatine-Tris, 2 MgATP, and 0.5 Na_3_GTP; pH 7.2–7.4, 280–285 mOsm). GFP-positive cells in the dorsal CA1 region of HPC were visualized using an upright microscope (Olympus) using infrared and epifluorescent illumination. CA1 pyramidal cells were distinguished by location in the cell body layer and individual cell morphology. Whole-cell patch-clamp recordings were made from transfected cells using a Multiclamp 700B amplifier and a Digidata 1440A digitizer (Molecular Devices), and whole-cell junction potential was not corrected. Traces were sampled (10 kHz), filtered (10 kHz), and digitally stored. Cells with membrane potential more positive than −50 mV or series resistance of >20 MΩ were omitted from analysis. Series resistance was measured as the sum of the access resistance (MΩ) and the tip resistance (MΩ).

Membrane capacitance, membrane resistance, and access resistance were measured according to the pClamp 10 manual (Molecular Devices). Briefly, the membrane capacitance, membrane resistance, and access resistance were determined by fitting the transient portion of the current response in the pClamp membrane test to an exponential function. Integrating this function yielded the area under the curve of the current response, and its components were automatically analyzed to determine the above membrane parameters. Input resistance was measured in Clampfit (Molecular Devices) from a current clamp input–output curve as the slope of the line fitted to the voltages recorded at increasing current injections (first voltage, <120 mV). Resting membrane potential (in mV) was read using the Multiclamp 700B Commander (Molecular Devices) while injecting no current (*I* = 0) immediately after breaking into a cell to avoid the error generated by the voltage drop across the resistance of the recording electrode during current injection. Bridge-balance compensation was not used in the above determination of membrane parameters.

Rheobase was measured by giving brief (250 ms) depolarizing (5 pA) steps with 250 ms between steps. The elicited action potential (AP) number was measured by issuing increasing depolarizing steps (25–300 pA, 500 ms) with 500 ms in between steps. For measures of hyperpolarization-activated cation current (*I*_h_), hyperpolarizing steps (500 ms) that produced potentials at −130 to −50 mV were issued in between the holding current. For synaptic recordings of spontaneous EPSCs (sEPSCs), cells were held at −80 mV for 2 min. Evoked EPSCs were conducted at −70 and +40 mV using a digital stimulator (Digitimer) and a tungsten stimulating electrode (50–500 µA, 50 µs; World Precision Instruments) placed locally in the stratum radiatum layer of CA1 to target Schaffer collateral fibers. All electrophysiology recordings were made at ∼30–32 °C by warming the aCSF line with a single inline heater (Warner Instruments).

### Data analysis

All traces were analyzed using pClamp (Clampfit; RRID:SCR_011323). Compiled data were analyzed using mixed two-way repeated-measures ANOVAs or one-way ANOVAs followed by Holm–Sidak-corrected multiple comparisons. Rheobase was taken as the lowest step necessary to elicit a spike. Spike frequency adaptation was measured from elicited action potentials at the maximal current step as the interspike interval (ISI; in ms). For measures of *I*_h_, peak and steady-state (S.S.) potential were obtained from the antipeak and for the last 20 ms of the step, respectively. *I*_h_ was measured at the maximal negative current necessary to hyperpolarize the cell to −120 mV. For sEPSCs, measurements across a 2 min trace were analyzed by Mini Analysis (Synaptosoft) and filtered with a low-pass Butterworth filter (500 Hz). sEPSCs with peak amplitudes not readily distinguishable from noise (≤5 pA) were excluded from the analysis. For AMPA/NMDA ratio, data for each cell were averaged from 10 evoked EPSCs. For EPSCs at −70 mV, peak amplitude was measured. For EPSCs at +40 mV, amplitude was measured 40 ms after stimulation to isolate NMDA current from the more transient AMPA current. Cells with low peak amplitude AMPA EPSCs (<100 pA at highest stimulus intensity) and/or low amplitude NMDA EPSCs (<100 pA at 50 ms after highest stimulus intensity) were excluded from the analysis. NMDA decay tau was determined based on previously established methods (Cathala et al., 2000). We averaged NMDA EPSCs (10–15 traces) and fitted the decay portion based on the following equation:

$$A\left(t\right)=A_{slow}*e^{-t/}{{}^{tslow}}_{}+A_{fast}*e^{-t/}{}^{tfast},$$

where τ_slow_ and τ_fast_ represent the decay time constants and *A*_slow_ + *A*_fast_ represent the amplitudes of the slow and fast components. We then calculated the weighted time constant (τ_w_) based on the following equation:

$$t_{w}=tt_{slow}*[A_{slow}/\left(A_{slow}+A_{fast}\right]+t_{fast}*\left[A_{fast}/\left(A_{slow}+A_{fast}\right)\right].$$

## Results

### ΔFosB decreases intrinsic membrane excitability of CA1 neurons

**Figure 1.**
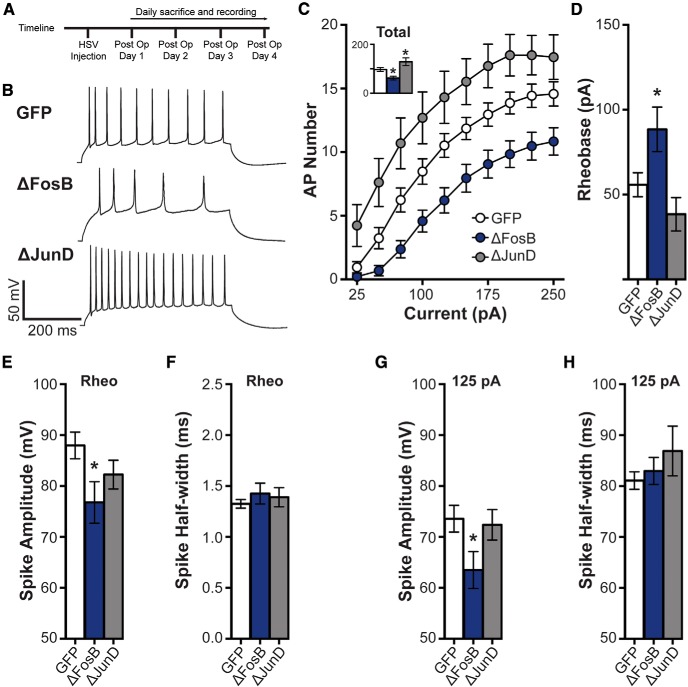
ΔFosB regulates spike number in hippocampal CA1 neurons. ***A***, Experimental timeline. ***B***, Representative spikes from 125 pA depolarizing current injection in dHPC CA1 neurons transduced by HSV-GFP (GFP), HSV-GFP+ΔFosB (ΔFosB), and HSV-GFP + ΔJunD (ΔJunD). ***C***, Spike (AP) number across increasing depolarizing current steps (25–250 pA) for GFP (*n* = 21 cells; *n* = 8 mice), ΔFosB (*n* = 19 cells; *n* = 9 mice), and ΔJunD (*n* = 14 cells; *n* = 5 mice). The number of APs was significantly decreased by ΔFosB, and increased by ΔJunD. *p < 0.05, **p < 0.01 (two-way repeated-measures ANOVA; Holm–Sidak comparisons). Inset, Total number of APs across all steps. Total spikes were similarly regulated by ΔFosB. *p < 0.05 (one-way ANOVA; Holm–Sidak comparisons). ***D***, Rheobase (in pA) in GFP-, ΔFosB-, and ΔJunD-expressing cells. Rheobase was significantly increased by ΔFosB expression. *p < 0.05 (one-way ANOVA; Holm–Sidak comparisons). ***E***, Rheobase spike peak amplitude (in mV). Spike amplitude was significantly decreased by ΔFosB expression. *p < 0.05 (one-way ANOVA; Holm–Sidak comparisons). ***F***, Rheobase spike half-width (in ms). No differences were observed. ***G***, Mean spike amplitude (in mV) elicited by 125 pA depolarizing current injection. Spike amplitudes at 125 pA current were significantly decreased by ΔFosB expression. *p < 0.05 (one-way ANOVA; Holm–Sidak comparisons). ***H***, Mean spike half-width (in ms) elicited by 125 pA depolarizing current injection. No differences were observed. All graphs display means ± SEM.

To determine the role of ΔFosB in regulating dHPC CA1 pyramidal cell function, we examined the effects of viral-mediated overexpression and inhibition of ΔFosB on intrinsic excitability and measures of synaptic strength (Fig. 1*A*). We first tested whether HSV-mediated overexpression of GFP and ΔFosB, or ΔJunD, an inhibitor of ΔFosB transcriptional regulation, affected basic cell and membrane properties. No changes were observed in resting membrane potential, membrane resistance, access (series) resistance, membrane capacitance, or input resistance (Table 1).

**Table 1:** Effects of ΔFosB overexpression or inhibition on properties of dorsal CA1 neurons.

Cell property	GFP (*n* = 21)	ΔFosB (*n* = 19)	ΔJunD (n = 14)
Resting potential (mV)	−66.7 ± 0.7	−67.9 ± 1.1	−66.3 -14.4
Membrane capacitance (pF)	71.6 ± 4.6	66.4 ± 5.4	60.0 ± 5.9
Membrane resistance (MΩ)	66.1 ± 9.0	58.9 ± 10.4	67.9 ± 7.9
Access resistance (MΩ)	18.7 ± 1.2	19.3 ± 1.3	18.7 ± 1.9
Input resistance (MΩ)	156.7 ± 11.9	171.9 ± 25.6	176.6 ± 15.2

Values are reported as the mean ± SEM.

To measure excitability, CA1 pyramidal cells were injected with increasing steps (500 ms) of current (Δ25 pA/step) with periods of rest (0 pA, 500 ms) in between the steps. Representative voltage traces to a 125 pA current step are shown in Figure 1*B*. ΔFosB overexpression decreased the number of spikes observed across steps (Fig. 1*C*; main effect of group: *F*_(2,51)_ =10.12, *p* < 0.001; Fig. 1*C*, inset: GFP vs ΔFosB, *p* = 0.024, 75–250 pA: *p* < 0.05 compared with GFP). No differences were observed in spike amplitude or half-width (data not shown), indicating that ΔFosB did not modify action potential kinetics. ΔFosB also increased the threshold necessary to elicit an AP (rheobase) in response to a brief depolarizing step of current (Fig. 1*D*; *F*_(2,51)_ = 5.73, *p* = 0.006; *p* = 0.044 compared with GFP).

Additionally, ΔFosB antagonism exerted an opposite effect, increasing excitability. ΔJunD, a dominant negative mutant of the binding partner of ΔFosB, JunD, which binds to endogenous ΔFosB and antagonizes transcriptional activity by preventing transactivation (Peakman et al., 2003), caused a persistent increase in AP number across depolarizing steps (Fig. 1*C*, inset: GFP vs ΔJunD, *p* = 0.035; Fig. 1*C*: 75–250 pA, *p* < 0.05 compared with GFP), while having no effect on rheobase (Fig. 1*D*). These findings suggest that endogenous ΔFosB decreases the intrinsic membrane excitability of dHPC CA1 neurons.

### Spike amplitude is decreased by ΔFosB expression

We next assessed the properties of the action potential: peak amplitude (in mV) and duration (half-width, in ms). We examined spike properties at both rheobase and the average spike properties of all spikes elicited by a 125 pA current injection (as shown in the representative traces in Fig. 1*B*). Spike amplitude at rheobase was significantly decreased by ΔFosB expression (Fig. 1*E*; one-way ANOVA: *F*_(2,50)_ = 3.26, *p* = 0.047; *post hoc*: *p* = 0.028), and this was also observed at the 125 pA current step (Fig. 1*G*; one-way ANOVA: *F*_(2,49)_ = 3.25, *p* = 0.047; *post hoc*: *p* = 0.039). ΔJunD expression did not significantly alter spike amplitude. In addition, no differences were observed in spike duration at rheobase (Fig. 1*F*) or 125 pA current (Fig. 1*G*). Thus, ΔFosB overexpression in CA1 neurons appears to decrease the peak amplitude of action potentials.

### Spike frequency adaptation is enhanced by ΔFosB expression

**Figure 2.**
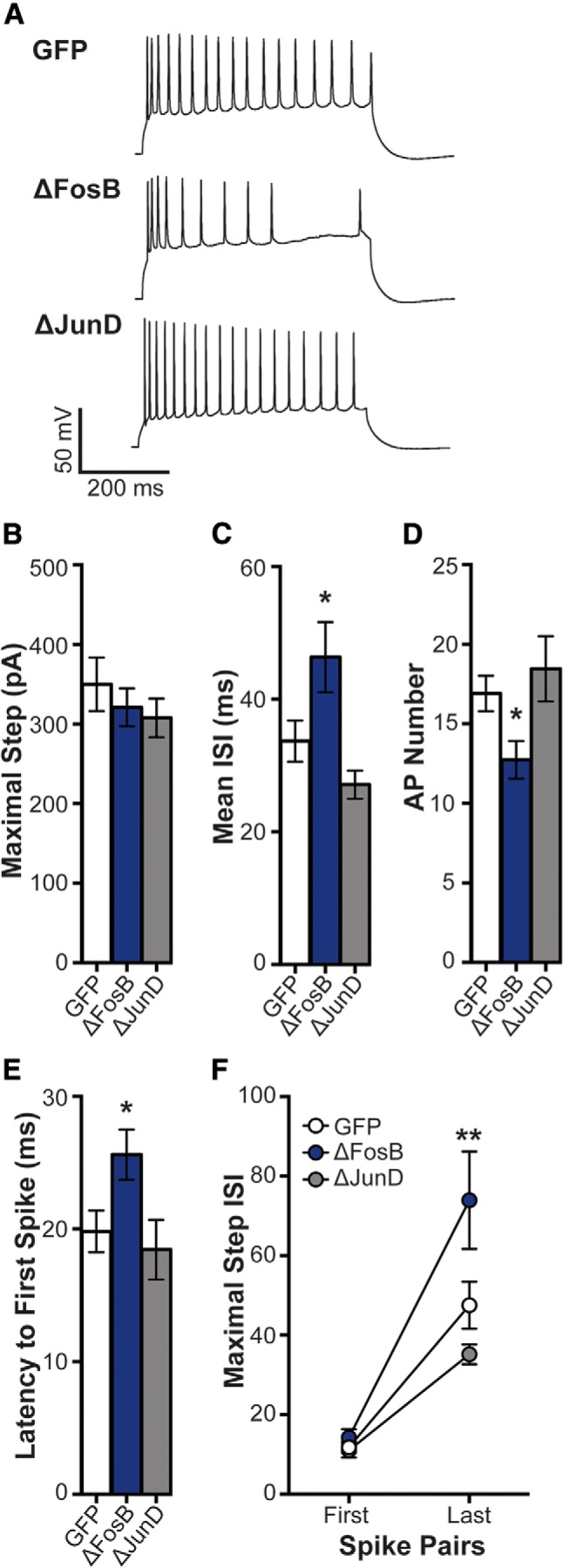
ΔFosB enhances spike frequency adaptation. ***A***, Representative spikes at maximal depolarizing current injection in dHPC CA1 neurons transduced by HSV-GFP (GFP), HSV-GFP + ΔFosB (ΔFosB), and HSV-GFP + ΔJunD (ΔJunD). ***B***, The maximal depolarizing current was used to assess spike frequency adaptation among GFP (*n* = 21 cells; *n* = 8 mice), ΔFosB (*n* = 19 cells; *n* = 9 mice), and ΔJunD (*n* = 14 cells; *n* = 5 mice). No differences were observed in maximal current; *p* > 0.05 (one-way ANOVA). ***C***, Mean ISI (in ms) at the maximal step across groups. Mean ISI was increased by ΔFosB; *p < 0.05 compared with GFP (one-way ANOVA; Holm–Sidak comparisons). ***D***, Number of APs at the maximal step across groups. ΔFosB significantly decreased the number APs; *p < 0.05 compared with GFP (one-way ANOVA; Holm–Sidak comparisons). ***E***, Latency from the onset of the maximal step and the first spike was assessed for each group. ΔFosB increased the latency compared with GFP; *p < 0.05 (one-way ANOVA; Holm–Sidak comparisons). ***F***, ISI for the first and last pair of spikes at the maximal step for each group. ISI at the last pair of spikes was increased by ΔFosB compared with GFP; **p < 0.01 (two-way repeated-measures ANOVA; Holm–Sidak comparisons). All graphs display means ± SEM.

Excitability may reflect changes in spike frequency adaptation as a result of differential ion channel expression (Storm, 1990; Fleidervish et al., 1996; Gu et al., 2007). Thus, ΔFosB may mediate intrinsic mechanisms (e.g., ion channel expression and/or function), which influence cellular excitability in CA1 neurons by increasing spike adaptation across depolarizing current or by changes in hyperpolarization-activated current. Therefore, we investigated spike adaptation (measured by ISI) in elicited APs at the maximal depolarizing current step in GFP-, GFP + ΔFosB-, and GFP + ΔJunD-expressing CA1 neurons. Groups did not differ in their maximal depolarizing step current (Fig. 2*A*). At this step, ΔFosB overexpression increased mean ISI (Fig. 2*B*; one-way ANOVA: *F*_(2,48)_ = 5.73, *p* = 0.006; *post hoc*: *p* = 0.039), decreased AP number (Fig. 2*C*; one-way ANOVA: *F*_(2,50)_ = 4.60, *p* = 0.015; *post hoc*: *p* = 0.048), and increased the latency from the onset of the step and the first spike (Fig. 2*D*; one-way ANOVA: *F*_(2,48)_ = 4.06, *p* = 0.023; *post hoc*: *p* = 0.047). Therefore, the maximal step was determined to be the ideal current to investigate whether the overall decrease in spike frequency by ΔFosB overexpression was due to altered spike adaptation.

We next assessed the ISI for the first and last spike pairs at the maximal step (Fig. 2*E*). All groups increased ISI at the last spike pair compared with the first spike pair (main effect of spike pair: *F*_(1,48)_ = 75.43, *p* < 0.001). No differences were found between groups at the first pair of spikes; however, ΔFosB significantly increased ISI at the last pair of spikes (interaction of ${}^{\text{spike}\;\text{pair}}{\text{X}}^{\text{group}}$: *F*_(2,48)_ = 4.95, *p* = 0.011; *post hoc*: *p* = 0.003 compared with GFP). This suggests that the ΔFosB reduction of CA1 neuron excitability may be due to an enhancement in spike frequency adaptation.

### ΔFosB overexpression decreases hyperpolarization-activated current in CA1 neurons

**Figure 3.**
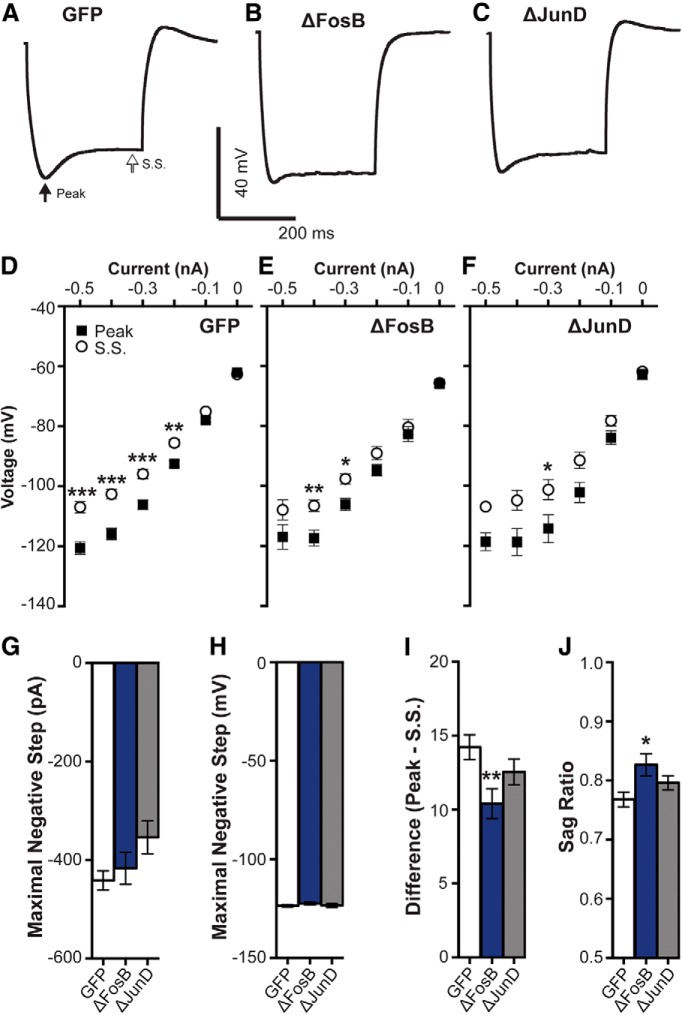
ΔFosB decreases *I*_h_. ***A–C***, Representative voltage trace to a hyperpolarizing current step showing *I*_h_ in hippocampal CA1 neurons expressing GFP (*n* = 21 cellls; *n* = 8 mice), ΔFosB (*n* = 19 cells; *n* = 9 mice), and ΔJunD (*n* = 13 cells; *n* = 5 mice), respectively. Arrows indicate amplitude measured at peak and S.S. ***D−F***, *I–V* curves from hyperpolarizing current injections (0 to −500 pA) at peak and S.S. in GFP, ΔFosB, and ΔJunD cells. GFP cells had significant differences between peak and S.S. across currents (from −500 to −200 pA), indicative of *I*_h_. ***p < 0.001 (two-way ANOVA; Holm–Sidak comparisons). Differences between peak and S.S. were comparably reduced in both ΔFosB (only different at steps −400 and −300 pA) and ΔJunD groups (only at −300 pA), indicative of reduced *I*_h_. *p < 0.05, **p < 0.01 (two-way ANOVAs; Holm–Sidak comparisons). ***G***, ***H***, Mean maximal negative current and voltage (produced by maximal negative current), respectively, in GFP, ΔFosB, and ΔJunD cells. No differences were observed between groups; *p* > 0.05 (one-way ANOVA). ***I***, Difference between peak and S.S. at maximal negative current across groups. ΔFosB significantly decreased the difference compared with GFP; **p < 0.01 (one-way ANOVA; Holm–Sidak comparisons). ***J***, Sag ratio (S.S./peak at maximal negative current ratio) across groups. ΔFosB significantly increased the sag ratio (decreased *I*_h_) compared with GFP; *p < 0.05 (one-way ANOVA; Holm–Sidak comparisons). All graphs display means ± SEM.

Hippocampal neuron activity, like that produced by theta burst stimulation, can produce decreases in cellular, but not dendritic, excitability that are associated with increased *I*_h_ (Fan et al., 2005). Therefore, we also examined the effects of ΔFosB on *I*_h_ in dHPC CA1 neurons. Representative hyperpolarizing voltage traces displaying peak voltage and S.S. for CA1 cells expressing GFP, GFP + ΔFosB, and GFP + ΔJunD are shown in Figure 3*A–C*. Across hyperpolarizing steps, GFP cells show voltage differences between peak and S.S. (Fig. 3*D*; Interaction of ${}^{\text{state}}{\text{X}}^{\text{step}}$: *F*_(5,204)_ = 8.98, *p* < 0.001; *p* < 0.001 between peak and S.S. across −500 to −200 pA hyperpolarizing steps), indicative of *I*_h_ that is normally observed in CA1 pyramidal neurons (Maccaferri et al., 1993; Magee, 1999). However, ΔFosB decreased these differences across current steps (Fig. 3*E*; *p* > 0.05 for interaction of ${}^{\text{state}}{\text{X}}^{\text{step}}$; *p* < 0.05 between peak and S.S. at only −400 and −300 hyperpolarizing steps). The antagonism of ΔFosB by ΔJunD may have also mildly reduced *I*_h_ compared with GFP, indicated by lower mean differences between peak and S.S. across hyperpolarizing steps (Fig. 3*F*, bottom; *p* > 0.05 for interaction of ${}^{\text{state}}{\text{X}}^{\text{step}}$; *p* < 0.05 between peak and S.S. across −400 to −200 pA hyperpolarizing steps).

We also examined I_h_ at the maximal negative current step (see Materials and Methods), which did not differ between groups (Fig. 3*G*,*H*; *p* > 0.05). At this step, ΔFosB significantly reduced the difference between peak and S.S. (Fig. 3*I*; one-way ANOVA: *F*_(2,49)_ = 4.83, *p* = 0.012; *post hoc*: *p* = 0.006 compared with GFP) and the sag ratio (calculated as S.S./peak; Fig. 3*J*; one-way ANOVA: *F*_(2,49)_ = 4.17, *p* = 0.021; *post hoc*: *p* = 0.012 compared with GFP). These findings suggest that I_h_ is regulated by ΔFosB, which may contribute to the change in excitability; however, the blockade of ΔFosB does not appear to sufficiently alter basal I_h_ values in CA1 neurons to account for the increased excitability observed in Figure 1. This, along with the finding that ΔJunD does not regulate spike adaptation, suggests that enhanced excitability caused by ΔFosB antagonism may be driven by different mechanisms than spike adaptation or I_h_.

### ΔFosB alters synaptic strength in CA1 neurons

**Figure 4.**
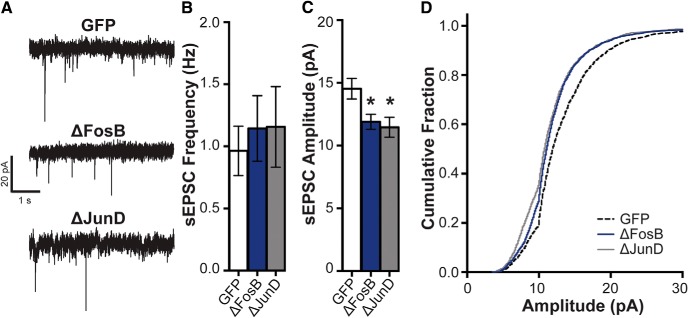
ΔFosB decreases spontaneous EPSC amplitude. ***A***, Representative traces (−80 mV) showing sEPSCs in hippocampal CA1 neurons expressing GFP (*n* = 20 cells; *n* = 7 mice), ΔFosB (*n* = 16 cells; *n* = 6 mice), and ΔJunD (*n* = 9 cells; *n* = 5 mice). ***B***, sEPSC frequency (in Hz) across groups. No differences in sEPSC frequency were observed. ***C***, sEPSC peak amplitude (in pA) across groups. Both ΔFosB and ΔJunD reduced sEPSC amplitude compared with GFP; *p < 0.05 (one-way ANOVA; Holm–Sidak comparisons). ***D***, Cumulative fraction of the peak amplitudes (5–30 pA) across groups. Both ΔFosB and ΔJunD were shifted to the left from GFP. All graphs display means ± SEM.

ΔFosB increases dendritic spine formation in NAc and dHPC, and produces cell type-specific adaptations in synaptic strength in NAc MSNs (Grueter et al., 2013; Eagle et al., 2015). This suggests that ΔFosB may also regulate functional synaptic plasticity in dHPC CA1 neurons. Therefore, we sought to assess the role of ΔFosB in sEPSCs and AMPA/NMDA ratios in CA1 neurons. sEPSCs were assessed at −80 mV over 2 min periods. Representative traces of sEPSCs are shown in Figure 4*A*. No changes were observed in the sEPSC frequency (Fig. 4*B*), but both ΔFosB and ΔJunD decreased sEPSC amplitudes (Fig. 4*C*,*D*; *F*_(2,42)_ = 4.75, *p* = 0.014; *p* = 0.027 for ΔFosB and *p* = 0.027 for ΔJunD compared with GFP, respectively), suggesting that both ΔFosB overexpression and antagonism may reduce the postsynaptic response in CA1 neurons. Because sEPSCs are generated by AP-dependent and AP-independent release of neurotransmitter in the absence of experimental stimulation, they cannot conclusively implicate either presynaptic or postsynaptic changes produced by ΔFosB modification in CA1 neurons.

**Figure 5.**
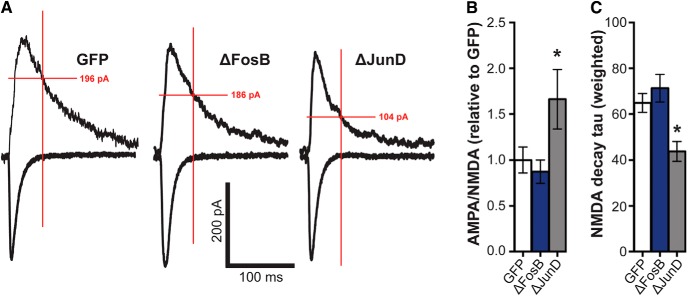
Blockade of ΔFosB enhances AMPA/NMDA ratio. ***A***, Representative average number of evoked EPSCs at −70 and +40 mV in dHPC CA1 neurons expressing GFP (*n* = 19 cells; *n* = 8 mice), ΔFosB (*n* = 13 cells; *n* = 9 mice), and ΔJunD (*n* = 10 cells; *n* = 4 mice). Red lines indicate the measurement for NMDA currents, 50 ms after stimulation. ***B***, AMPA/NMDA ratios (normalized relative to GFP) across groups. ΔJunD significantly increased the AMPA/NMDA ratio compared with GFP. ***C***, Decay tau for NMDA currents (weighted across both fast and slow components) across groups. ΔJunD significantly reduced NMDA decay tau; *p < 0.05 (one-way ANOVA; Holm–Sidak comparisons). All graphs display means ± SEM.

To directly assess whether ΔFosB regulates postsynaptic strength in CA1 neurons, we examined glutamate receptor function using AMPA/NMDA ratios of evoked EPSCs. Representative traces of evoked EPSCs at −70 and +40 mV are shown in Figure 5*A*. Interestingly, ΔJunD-mediated antagonism of ΔFosB significantly increased the AMPA/NMDA ratio (Fig. 5*B*; *F*_(2,39)_ = 4.41, *p* = 0.023; *p* = 0.038 compared with GFP), but no differences were found by ΔFosB overexpression. This ΔJunD-driven increase in AMPA/NMDA ratio, which is usually interpreted as an increase in synaptic strength, is in stark contrast to the ΔJunD-driven decrease in sEPSC amplitude (Fig. 4). Therefore, we also assessed the kinetic properties of the evoked EPSCs. We did not detect any differences in the AMPA-mediated amplitude of evoked EPSCs at −70 mv; however, the decay time constant of the NMDA-mediated current was significantly reduced by ΔJunD overexpression (Fig. 5*C*; *F*_(2,39)_ = 6.87, *p* = 0.003; *p* = 0.011 compared with GFP). This suggests that antagonism of ΔFosB produces a decreased NMDA current, potentially via changes in receptor subunit composition, leading to the apparent increase in AMPA/NMDA ratios.

## Discussion

We demonstrate that ΔFosB overexpression decreases the excitability of dHPC CA1 pyramidal neurons, and, conversely, antagonism of endogenous ΔFosB function increases excitability. This is the first study to suggest that an activity-dependent transcription factor decreases excitability in these cells and raises the question of whether this is mediated by direct transcriptional regulation of ion channel expression or via regulation of other intermediary signaling or transcriptional regulators, such as the ΔFosB targets CaMKII or c-Fos (Robison et al., 2013, 2014; You et al., 2017). ΔFosB could be directly downregulating the expression of specific Na^+^ channels, or could achieve their downregulation indirectly by repression of immediate early genes, such as c-*fos*, which regulate Na^+^ channel expression. For example, ΔFosB represses c-*fos* expression via histone deacetylation (Renthal et al., 2008; You et al., 2017), which has been observed recently in dentate gyrus hippocampal neurons (Corbett et al., 2017). Alternatively, ΔFosB may be enhancing the expression or function of ion channels associated with decreased excitability (e.g., K^+^ channels). However, target genes of ΔFosB in hippocampus have not yet been fully delineated.

Homeostatic changes in excitability are associated with enhanced synaptic strengthening, typically after repeated neuronal activity or learning (Desai et al., 1999; Zhang and Linden, 2003), and this is regulated by both transcriptional and epigenetic mechanisms (Schulz et al., 2008; Guzman-Karlsson et al., 2014; Meadows et al., 2015, 2016). Thus, the reduction in excitability may be a compensatory cellular mechanism resulting from enhanced dendritic or synaptic activity, although future studies are needed to fully determine the role of ΔFosB in synaptic plasticity. However, this idea raises interesting possibilities that the ΔFosB-driven reduction in excitability may contribute to homeostatic mechanisms producing decreased global activity, thereby enabling the fine tuning of synapses after experience. Furthermore, the role of hippocampal ΔFosB in learning (Eagle et al., 2015; Corbett et al., 2017) indicates that this may be linked to experience-driven changes underlying memory consolidation. Excitability was recently hypothesized to be linked to place cell selectivity and memory allocation (Schmidt-Hieber and Nolan, 2017). In this hypothesis, increased excitability distinguishes place cells amid pools of “silent” non-place cells. Therefore, ΔFosB induction and its subsequent effects on excitability may act as a mechanism enhancing signal-to-noise in the pool of place-to-silent cells. Additionally, potential fine tuning of hippocampal plasticity of ΔFosB, both intrinsic and synaptic, may be aberrant in neurologic diseases, such as Alzheimer’s disease and epilepsy, and contribute to cognitive dysfunction (Corbett et al., 2017; You et al., 2017).

We also demonstrated that the decrease in excitability was associated with a concurrent decrease in I_h_ and an increase in spike frequency adaptation. I_h_ regulates excitability (van Welie et al., 2004; Fan et al., 2005; Kim Chung et al., 2012), primarily by homeostatically decreasing excitability after synaptic strengthening from neuronal activity (van Welie et al., 2004; Brager and Johnston, 2007). Therefore, these findings indicate that I_h_ may be contributing to the ΔFosB-mediated change in excitability. However, because I_h_ is typically associated with an increase in excitability (Fan et al., 2005; Kim Chung et al., 2012), the decreased I_h_ we observe may not be causally related to the change in excitability and may reflect ΔFosB-mediated transcription of targets that separately reduce both excitability and I_h_. Future studies using pharmacological isolation and blockade of I_h_ will be needed to determine the relationship between the effects of ΔFosB on I_h_ and excitability.

It is also possible that the reduction in excitability is driven by a ΔFosB-mediated increase in spike frequency adaptation. Spike adaptation is typically regulated by M-type K^+^ currents or inactivation of depolarizing currents (Storm, 1990; Benda and Herz, 2003; Otto et al., 2006; Gu et al., 2007). Additionally, the concurrent change in excitability and spike frequency adaptation may be driven by changes in membrane voltage (Fernandez et al., 2011), but this is unlikely as we did not observe a change in resting membrane potential. ΔFosB may therefore be transcriptionally activating or silencing targets that regulate spike adaptation (i.e., Na^+^ or K^+^ channels), which may, in part, underlie the decrease in excitability. During a depolarization, Na^+^ channels are inactivated and recover only gradually, contributing to spike adaption and a decrease in spike amplitude (Benda and Herz, 2003). ΔFosB could be acting to either prematurely inactivate Na^+^ channels or depress the recovery of Na^+^ channels. This potential mechanism would support a decrease in spike adaptation as well as the decrease in spike amplitude. These findings collectively point to ΔFosB regulating intrinsic membrane excitability in CA1 neurons, and future studies will uncover the specific transcriptional targets and downstream mechanisms driving this phenomenon.

The finding that ΔJunD antagonism of ΔFosB increased AMPA/NMDA ratios while decreasing sEPSC amplitude appears contradictory at first pass; however, this change in AMPA/NMDA ratio is likely explained by a decreased NMDA-mediated current rather than an increased AMPA current. NMDAR decay kinetics are determined in part by receptor subunit composition, with GluN2A-containing receptors having much faster decay than GluN2B- or GluN2C-containing receptors (Vicini et al., 1998). It is interesting to speculate that ΔFosB may be regulating the expression of the *GRIN2A* or *GRIN2B* genes in hippocampal CA1 neurons, a function that ΔFosB does not appear to have in NAc MSNs (Kelz et al., 1999; Robison et al., 2013). Future experiments examining ΔFosB gene targets in hippocampal pyramidal neurons, potentially including chromatin immunoprecipitation and gene expression studies, will be required to determine whether NMDAR subunit expression is indeed directly regulated by ΔFosB.

Together, the decreased sEPSC amplitude and NMDA-mediated current makes it likely that antagonism of ΔFosB leads to decreased synaptic strength. ΔJunD has been previously identified to decrease the total number as well as the number of mature, mushroom-shaped dendritic spines in dorsal CA1 neurons (Eagle et al., 2015), a structural phenomenon associated with reduced synaptic strength (Ziv and Smith, 1996; Engert and Bonhoeffer, 1999; Toni et al., 2007). Changes in synaptic strength in dorsal CA1, such as decreased NMDAR function and/or expression, are associated with a variety of impairments in learning and memory (McHugh et al., 1996; Tsien et al., 1996; Wilson and Tonegawa, 1997). Supporting this, ΔFosB antagonism by ΔJunD is known to impair spatial and contextual learning (Eagle et al., 2015). Thus, the current findings suggest that ΔFosB transcriptional activity is necessary for dorsal CA1-mediated synaptic plasticity underlying memory consolidation, potentially via direct action on NMDAR gene expression.

In addition to learning and memory, ΔFosB is also robustly induced in hippocampus by antidepressants, such as fluoxetine (Vialou et al., 2015), suggesting that it may underlie resilience to stress and depressive phenotypes. These novel findings suggest that a potential mechanism for antidepressant effects may be a decrease in the excitability of hippocampal neurons. Antidepressants, particularly selective serotonin reuptake inhibitors, have anticonvulsant properties (Yan et al., 1994; Favale et al., 1995; Wada et al., 1995), which may be mediated by decreased excitability through the inhibition of various Ca^2+^ and/or K^+^ channels (Deák et al., 2000; Choi et al., 2004; Igelström and Heyward, 2012) either indirectly or via serotonergic mechanisms (Andrade, 2011). Similarly, ΔFosB is also induced in HPC by chronic exposure to drugs of abuse (Perrotti et al., 2008), and it has been found that chronic amphetamine use reduces the intrinsic excitability of ventral subiculum neurons (Cooper et al., 2003). Therefore, ΔFosB may act as a critical regulator of general hippocampal excitability under a variety of disease conditions, from depression to addiction, or in specific ensembles of CA1 neurons during learning. Future studies will determine the gene targets and downstream mechanisms of these effects and delineate their possible roles in both normal and aberrant hippocampal function.

## Synthesis

Reviewing Editor: Douglas Bayliss, University of Virginia School of Medicine Decisions are customarily a result of the Reviewing Editor and the peer reviewers coming together and discussing their recommendations until a consensus is reached. When revisions are invited, a fact-based synthesis statement explaining their decision and outlining what is needed to prepare a revision will be listed below. The following reviewer(s) agreed to reveal their identity: Matthew Hearing.

This manuscript was seen by two reviewers, both of whom found that the results were important in delineating a link between expression of an activity-dependent transcription factor and neuronal excitability, with implications for plasticity mechanisms. However, as you will see from their comments (provided below), there were a number of concerns regarding statistical analysis, data presentation and interpretation that should be addressed. Of particular note, both reviewers shared concerns regarding the presynaptic mechanism that was suggested for effects on spontaneous and evoked EPSCs. This point should be clarified in a revised manuscript by including additional experiments/analysis suggested in the reviewer comments (e.g., paired pulse ratio, decay tau of NMDA current, etc.), and with further consideration of alternative interpretations. Full Reviews:

Reviewer #1

Advances the Field

This manuscript provides evidence for a link between an activity dependent transcription factor and functional modifications in neuronal function.

Abstract

It would be helpful in identifying JunD as an "antagonist" of FosB.

Comments to the Authors

In this study titled "deltaFosB decreases excitability of dorsal hippocampal CA1 neurons" the authors utilize viral-mediated overexpression to probe the consequences of the transcription factor deltaFosB on membrane and synaptic properties. Overall the manuscript is well designed and written. However, there a minor confound that needs to be addressed.

The authors had a very clear interpretation of the results except for the very interesting effects of deltaJunD on A/N ratio. The representative trace shows a clear difference in decay kinetics at the +40 dual EPSC. The T1/2 analysis of the decay may suggest a robust decrease in NMDA receptor mediated current. This would be consistent with an increase in A/N ratio that could overshadow the decrease in the AMPA receptor sEPSCs. The presynaptic explanation, although possible, is unlikely.

Reviewer #2

Advances the Field

The findings have important implications for a number of neurological disorders, as it is important to determine how common transcription factors that are so intricately linked with synaptic plasticity may have disociable (opposing) effects on cellular physiology that in turn have wide-reaching impacts on neural circuits. Further, if reviewer concerns are properly addressed, this work could expand upon mechanisms related to how deltaFosB in fact promotes plasticity.

Comments to the Authors

The authors examined the consequences of viral-mediated overexpression or inhibition of FosB on intrinsic physiological properties, as well as excitatory synaptic strength in adult mouse hippocampal CA1 pyramdial neurons using ex vivo whole-cell electrophysiological techniques. They found that overexpression of FosB decreased excitability and reduced Ih currents, while inhibition increased excitability of these neurons. Neither manipulation impacted resting membrane potentials or current-elicited spike firing, however FosB cells did show reduced Ih currents, which is particualry interesting as increased Ih currents are more often associated reduced excitability. While both FosB and JunD decreased sEPSCs, but only inhibition of FosB (JunD cells) altered synaptic strength measured by changes in AMPAR:NMDAR ratios - albeit in an opposing direction. Overall the manuscript was very well written, however electrophysiology method descriptions need bolstering (see comments below). The findings have important implications for a number of neurological disorders, as it is important to determine how common transcription factors that are so intricately linked with synaptic plasticity in other brain regions may impact cell types in others (i.e., nucleus accumbens versus the hippocampus).

Figure 1-2:

1. Although spike duration was assessed and did not appear to be altered, the authors should consider examining spike amplitude, as representative traces suggest that deltaFosB may be reducing the amplitude, and perhaps deltaJunB increasing it compared to GFP controls.

2. In the results for Figure 1, a main effect of group is reported from a Two-way RM ANOVA analysis. If no interaction was obtained between current and treatment (group), how is it that the authors decided it was appropriate to identify comparisons across groups at individual current amplitudes? This seems inappropriate and should be adjusted. For rheobase comparison in these same cells, was deltaFosB and deltaJunD groups not compared? It seems like these would be worthwhile comparing even if it is not significantly reduced compared to GFP controls.

3. In figure 1B, are the representative traces all from the same current step amplitude? If so, it should be stated as such and at which amplitude.

4. It is unclear what the authors mean by maximum current step. Does this mean the max current prior to depolarization-induced inhibition occurs (i.e., AP number begins to decline)? This does not seem to be the case given that in figure 2 the mean max step is reported to be just below 200 pA, however in figure1, the number of APs is still rising on average in both these groups.

5. In Figure 1 illustrating spike generation as a function of depolarizing current injections, the slope fall three groups appears to be the same. Moreover, groups did not differ in max current, suggesting that the firing capacity at more depolarized potentials is not altered. For example, if firing capacity (which could also be viewed as a spike frequency adaptation) were enhanced, GFP cells would show lower max current step. Together, these data suggest that the differences in spike firing purely reflect differences in rheobase (i.e., left or right shift), whereby cells are firing more at a given amplitude for one group versus another primarily because they started firing sooner. While an alternative possibility is addressed by the authors in their examination of spike frequency adaptations, it makes the requests for methodological clarifications (see below) extremely important.

6. The authors state: "Therefore, the maximal step was determined to be the ideal current to investigate whether the overall decrease in spike frequency was due to spike adaptation." It is not clear exactly why this makes the max current ideal. Is it because this is where significant differences were found across all groups? It's concerning that a post hoc stategy was employed here, rather than ad hoc. One example is that if we assume the representative traces in figure 1 are from the same current amplitude, there appears to be a very distinct difference in the first ISI across groups (at the very least GFP/deltaJunD vs deltaFosB). Notably the increase in ISI is still proment in the FosB group in these representative traces, but a noticeable difference between JunD and FosB, and GFP vs. JunD may also be present a non-maximum steps (assuming these traces are non max). It would be helpful if the authors can provide more background on the validity of this approach based on previous literature examining ISI and spike adaptation. It would also be helpful if the traces in Figure 1 are not from max current steps that they provide representatives at max for figure 2.

Excitatory plasticity (Figures 4-5):

1. It would be helpful if the authors could clarify the justification for collecting sEPSCs for only 2 min, rather than ensuring that each recording utilizes a random stretch of an approximate number of events (e.g., 200-300). It is also unclear when this 2 min window occurred. Was this following a period of allowing the cell to stabilize? Further, the authors need to clarify why spontaneous and evoked EPSCs recorded at different potentials?

2. The authors indicate that the overall decrease in sEPSC amplitude in JunD neurons may be driven presynaptically. If not for the extremely odd (divergent) results across sEPSCs and A/N ratios, the authors need to directly examine this possibility as this is a particularly important point given that their methods selectively target postsynaptic cells and would greatly increase the impactfulness of this study if a presynaptic mechanism were shown. While removing the activity-dependent component with synaptic activity blockers would more readily isolate the postsynaptic component (mEPSCs) this still leaves the possibility of presynaptic components as increased frequency may reflect increased release probability. Thus a paired pulse ratio approach or somewhat equivalent approach such as coefficient of variance or variance mean analysis should be done (PPR would be the more straightforward).

3. The authors need to consider the possibility that discrepancies in JunD cells and FosB cells across these two assays may be due to changes in NMDARs. sEPSCs are collected at hyperpolarized potentials where NMDAR-mediate currents are virtually not present, however an increase in the ratio could reflect reductions in NMDAR currents or increases in AMPAR currents or both.

Along these lines, the authors need to clarify why are they measuring the NMDAR-current at 50 ms post stimulation. While isolating currents in this manner is done, it is not ideal, and is commonly done at 35-40 pA (as AMPAR-mediated components are generally not present following 30-35 ms)? The authors should consider using an NMDAR antagonist (D-APV) to pharmacologically isolate AMPAR-mediated currents and then digitally subtract out these isolated currents from the dual component current. At the very least the authors need to re-analyze using a more appropriate post-stimulation time point for NMDAR current, as differences in decay kinetics based on subunit composition (GluN2A vs 2B) may play an unwanted factor at a more protracted post-stim time point.

4. For evoked EPSCs (AMPAR:NMDAR ratios) was a K+ gluconate solution also used? IF not, the authors need to state which solution they used in the methods. If so, this is very unusual and not ideal, since the active conductance at the dendrite can affect the excitatory inputs on the postsynaptic neuron, which is why these recordings are done with cesium and QX-314 blocking K + and Na +, respectively. Moreover, while not absolutely necessary for measures of basal transmission, more stable recordings would be obtained using a similar solution for sEPSCs.

Discussion:

1. The authors state that reductions in sEPSC in FosB cells could reflect appearance of new immature (silent) synapses. There are at least 2 reasons why this statement seems inappropriate. First, this would likely be reflected at the level of sEPSC event frequency - which was not altered in FosB cells - rather than amplitude. Second, reductions in these measures would more likely reflect development of silent synapses due to endocytosis of AMPARs at previously non-silent synapses rather than synaptogenesis. The authors should adjust the text accordingly.
